# Description of the Hamburg Alexander Leukodystrophy Cohort—Insights into Practical Classification and the Care Situation

**DOI:** 10.3390/jcm14196918

**Published:** 2025-09-29

**Authors:** Nadia Kokaly, Helena Guerreiro, Janna Bredow, Steffi Dreha-Kulaczewski, Andreas Ohlenbusch, Wolfgang Köhler, Tabea Reinhardt, Gerhard Schön, Alexander E. Volk, Helen Sigel, Annette Bley

**Affiliations:** 1 University Children‘s Hospital, University Medical Center Hamburg-Eppendorf (UKE), 20246 Hamburg, Germany; 2 German Center for Child and Adolescent Health (DZKJ), Partner Site Hamburg, University Medical Center Hamburg-Eppendorf (UKE), 20246 Hamburg, Germany; 3 Faculty of Medicine and Biomedical Sciences, University of Algarve, 8005-139 Faro, Portugal; 4 Algarve Biomedical Center (ABC), 8005-139 Faro, Portugal; 5 Algarve Local Health Unit (ULS Algarve), 8000-386 Faro, Portugal; 6 Diagnostic and Interventional Neuroradiology, University Medical Center Hamburg-Eppendorf (UKE), 20246 Hamburg, Germany; 7 Department of Pediatrics and Adolescent Medicine, University Medical Center Göttingen, 37075 Göttingen, Germany; sdreha@gwdg.de (S.D.-K.); aohlenb@gwdg.de (A.O.); 8 German Center for Child and Adolescent Health (DZKJ), Partner Site Göttingen, University Medical Center Göttingen, 37075 Göttingen, Germany; 9 Leukodystrophy Outpatient Clinic, University of Leipzig Medical Center, 04109 Leipzig, Germany; wolfgang.koehler@medizin.uni-leipzig.de; 10 Center of Child and Youth Medicine, Klinikum Saarbrücken, 66119 Saarbrücken, Germany; treinhardt@klinikum-saarbruecken.de; 11 Center of Experimental Medicine, Institute for Medical Biometry and Epidemiology, University Medical Center Hamburg-Eppendorf (UKE), 20246 Hamburg, Germany; g.schoen@uke.de; 12 Institute of Human Genetics, University Medical Center Hamburg-Eppendorf (UKE), 20246 Hamburg, Germany; a.volk@uke.de

**Keywords:** Alexander disease, leukodystrophy, astrocytopathy, neurodegeneration, natural history, white matter, severity score, steroids

## Abstract

**Background**: Alexander disease (AxD) is a rare severe leukodystrophy that has no cure to date. A pathogenic gain-of-function variant in the *GFAP* gene affects the astrocytes and subsequently the function of the white matter in the CNS. **Methods**: We retrospectively analyzed the most frequent symptoms of nine AxD cases, classified them according to published classifications, and described the need of care and support. **Results**: The description of the courses of disease of nine cases with AxD reflects the broad spectrum of different phenotypes of AxD, with often occurring apnoea. Data about care and support for AxD patients indicate a high and heterogeneous need of support. Treatment with steroids reduced symptoms in two patients. Some patients showed lasting improvement during their course of disease. **Conclusions**: The course of AxD is very heterogeneous. Thus, we extracted relevant key features to describe the severity of the disease, namely feeding problems, epilepsy, age-appropriate motor function, failure to thrive, age-appropriate language and apnoea. We recommend early evaluation for clinical care and support. For some AxD patients, treatment with steroids may alleviate symptoms. Further development of efficient treatments is necessary.

## 1. Introduction

Alexander disease (AxD) was first described in 1949 by W. S. Alexander in a patient presenting with development delay, fretfulness, macrocephaly, and fibrinoid degeneration of fibrillary astrocytes [1].

Today, AxD is classified as a rare and progressive astrocytopathy, a subtype of leukodystrophy [2,3], caused by pathogenic variants in the *GFAP* (glial fibrillary acidic protein) gene on chromosome 17q21 [4,5]. The pathogenic gene variants are in most cases de novo missense variants, leading to a gain of function. AxD is inherited in an autosomal dominant manner [5,6,7]. GFAP is an intermediate filament protein, predominantly expressed in astrocytes in the CNS [5,8]. Astrocytes are a heterogeneous group of cells in the CNS, that influence the development and homeostasis in the brain as well as playing a role in the blood–brain barrier, myelination, the extracellular ionic milieu, the metabolic support of neurons, synaptic transmission, and neuronal plasticity, etc. [2].

The diagnosis of AxD is based on clinical symptoms and characteristic brain MRI findings. Until the description of the five typical MRI criteria [9] and identification of the underlying genetic defect in 2001 [5], diagnosis was confirmed by the proof of Rosenthal fibres (RF) in the histopathology of a brain biopsy [10,11]. RF are cell inclusions, consisting of different proteins including the overexpressed and accumulated intermediate filament GFAP as well as HSP27 and αB-crystalline [12,13], which are typical but not pathognomonic for AxD [14].

AxD is a progressive neurodegenerative disease, leading to multiple neurological disabilities including major affection of basic functions such as mobility, verbal communication, swallowing, cognitive functions, and breathing. Currently, there is no approved treatment available. Patients usually receive symptomatic treatment for the aforementioned problems, e.g., epilepsy, incontinence, constipation, as well as support for psychological and social issues [6]. Limited information exists regarding the actual care and support provided to patients with AxD. Therefore, this study reports on the disability status and the need for assistive devices among seven individuals diagnosed with AxD.

Recent studies with frequent intrathecal administration of a DNA-based antisense oligonucleotide (ASO) designed for degradation of GFAP mRNA are ongoing (NCT04849741) but are not yet available for most patients [15]. Some reports in the literature describe clinical improvement following steroid treatment [16,17], which led to individual therapeutic trials with steroids.

Here we report on the course of the disease in nine AxD patients. Over the last few years, different classifications of AxD, based on age of onset, MRI findings, and/or leading symptoms, have been published. We aimed for a better understanding of disease severity in our patients compared to other AxD patients and were interested in a better prediction of the clinical course. Hence, we classified nine AxD patients using the following four published classification systems: the traditional classification focusing on age of onset (neonatal, infantile, juvenile, adult) [6,18,19]; the classification by Yoshida et al. into cerebral, bulbospinal, or intermediate type [20]; the classification by Prust et al. into type 1 and 2 [21]; and the classification by Mura et al., focusing on the course of disease [22,23].

To cover the demand of capturing the great clinical variability of all patients we selected a small number of key features of AxD to develop a severity score that might allow a better grading and comparison of our patients within the broad spectrum of AxD. This also enabled us to rate our patients who presented with intermittent clinical symptom improvement despite subsequent phases of progressive deterioration.

## 2. Materials and Methods

Retrospective data from nine patients of the Hamburg leukodystrophy centre with a confirmed diagnosis of AxD, either by identification of a pathogenic variant GFAP or detection of Rosenthal fibres in autopsy, were analyzed. The study was approved by the Ethics Committee of the Medical Association of Hamburg (PV3782). All living patients or their caregivers gave informed consent for participation in this study. Retrospective data analysis was performed using medical records, including those from other medical centres involved in the patients’ care, and diagnostic findings, e.g., MRI results up to August 2023. The analysis focused on the symptoms, genetic findings, helpful treatment options, medical aids, and survival after diagnosis.

Percentiles were determined according to the KiGGS study by Kohse in 2014. Macrocephaly was defined as a head circumference at or above the 97th percentile and dystrophy was defined as a body weight at or below the 3rd percentile [24].

The genetic variants were described according to the HGVS nomenclature [25]. Known pathogenic variants were identified through a literature search, including the GFAP gene variant list of the Waisman Center [26,27], the public database ClinVar [28], and Human Gene Mutation Database (HGMD^®^) Professional. Novel GFAP variants were classified according to the recommendations of the ACMG by using Franklin by Qiagen/Genoox [29,30].

To assess the situation of care a questionnaire was completed for each patient at each visit. In this study, the most recent questionnaire was analyzed. Levels of care were described according to the German Sozialgesetzbuch (SGB)—Elftes Buch (XI)—Soziale Pflegeversicherung. The levels of care describe the degree of impairment, ranging from level 1, only mild impairment, these patients can provide for themselves, to level 5, most severe impairment, with special requirement for nursing care [31].

Classification was performed according to published classification systems:

1. Russo et al., 1976 [18], Springer et al., 2000 [19], Srivastava et al., 2002 [updated 2020] [6]: Traditional classification focusing on age of onset (neonatal, infantile, juvenile, adult).
2. Yoshida et al., 2011 [20]: Classification into three types (cerebral, bulbospinal, intermediate) as a guideline for diagnosis.
3. Prust et al., 2011 [21]: Classification into type 1 and 2.
4. Mura et al., 2021 [22], Vaia et al., 2023 [23]: Retrospective classification focusing on course of disease.

A sample size calculation for the ADSS was performed to determine the number of participants required for future validation studies, assuming clinically relevant differences of 2, 3, and 4 points.

## 3. Results

We present data of the course of disease of four male and five female patients with AxD. All nine patients were classified according to the published classification systems (see Supplementary Tables S1–S4). Table 1 provides an overview of diagnostic data and symptoms. Diagnosis of AxD was confirmed either by genetic testing or detection of Rosenthal fibres in autopsy.

### 3.1. Patients Case Reports

Patient 1 developed the first symptoms at the age of four months, presenting with a motor development delay primarily affecting the oral motor skills, resulting in an inability to eat with a spoon and a failure to thrive. The first seizure occurred at the age of one and a half years. The patient experienced various symptoms including pyramidal signs with motor deterioration (latest GMFCS level of V), bulbar symptoms, cognitive delay, sleep apnoea requiring a tracheostomy at five years of age, autonomic dysfunction, and paroxysmal deterioration.

Patient 2 presented the first symptoms at the age of six months, including a failure to thrive and developmental delay. The patient suffered from seizures and showed a motor, and later also cognitive, delay; macrocephaly; bulbar symptoms; and ataxia. The patient presented with typical MRI findings.

Patient 3 showed first symptoms at the age of four months, with a lack of head control and classic radiological features. Both led to the suspicion of AxD and the diagnosis was confirmed at the age of one year by genetic testing. The patient presented with various symptoms as seizures (the first one occurring at the age of 11 months), motor delay (GMFCS V), cognitive delay, macrocephaly, failure to thrive, paroxysmal deterioration, dysphagia, urinary and fecal incontinence, sleep disturbance, pyramidal signs, and chronic pain.

Patient 4 was described as a “bad eater” and presented with hypotonia but an otherwise normal motor development until the age of ten months. A progressive decline in motor abilities led to the necessity of a buggy at the age of nine years and a wheelchair at the age of 11 years. At the age of ten years a spastic ataxic gait disorder appeared. In addition to the motor delay, the patient presented with seizures (first one at the age of three and a half years), macrocephaly, failure to thrive, paroxysmal deterioration, dysphasia, slow speech, and dysphagia, leading to the necessity of a G-Tube. The patient also presented with autonomic dysfunction, sleep apnoea, pyramidal signs, hyperreflexia, and chronic pain, due to a progressive scoliosis. Typical radiologic features were found.

Patient 5 was initially classified as an atypical AxD case, presenting with an intention tremor of the hands and tremor of the head at the age of seven months as first symptoms. The disease progressed slowly, with intermittent deterioration. Typical MRI features were found. During the course of the disease the patient experienced episodes of vomiting and failure to thrive, as well as dysphagia and dysarthria, sleep apnoea, and respiratory insufficiency, leading to the necessity of resuscitation and ventilation on one occasion. A hemiparesis and subsequent falls led to the requirement of a wheelchair for one year. A treatment with steroids improved this situation significantly as well as the dysarthria and dysphagia. Dyscalculia, concentration problems, and spasticity occurred at the age of seven years. Epileptic seizures in form of absences were also treated with steroids and suspended under that therapy. Pyramidal signs and an ataxic gait disorder were reported. At the age of eight years, the head circumference was reported to be above the 99th percentile but normalized to the 81st percentile during further thriving. According to the caregivers, the steroid therapy trials led to an improvement of clinical symptoms and development. In the last examination, the patient presented only with minor symptoms and primarily fine motor difficulties but was able to walk unaided.

Patient 6 had an infantile onset of disease, manifesting as a gait ataxia at the age of one year and showed a prolonged progression. Subsequently, motor development was delayed and deteriorated. Muscle hypotonia, an intention tremor, and a cognitive delay beginning at school age were reported. Based on these symptoms, a suspected myopathy was initially diagnosed. At the age of eight years, brain imaging revealed diffuse global white matter lesions. Macrocephaly and psychiatric abnormalities occurred at the age of nine years. Gowers sign and pyramidal signs were positive. At that time the initial suspicion of a leukodystrophy, particularly AxD, was made. From the age of ten years, the patient was wheelchair-bound and a brain MRI revealed classical AxD findings. During the course of the disease, a bladder dyssynergy and a spastic paraparesis in the lower extremities occurred.

At the age of 16 years, the cognitive function worsened. Painful orthopedic problems were observed. Follow up data are incomplete up to the age of 32 years, but since the age of 21 years, signs of epileptic potentials in the EEG were seen and he was treated with levetiracetam. At the age of 25 years, an individual treatment attempt with dimethyl fumarate for approximately 1 year was made, based on the idea to reduce GFAP by Nrf2 activation, which had been analyzed in mouse models [14,32,33]. No deterioration was reported during this period, but the medication was discontinued due to recurrent infections.

The last report at the age of 32 years described a severely restricted and immobile patient, who could only perform minimal movements of the torso, shoulders, and hands. Symptoms included sleeping problems, daytime fatigue, absences, dysphagia, nystagmus in the gaze direction, bilateral positive Babinski sign, spasticity, and impaired breathing. Dysarthria was also severe, but the patient was still able to answer yes/no questions. A G-Tube for feeding was implemented; the patient was catheterized and used a cough assist as required.

Patient 7 had a late onset of disease; the first symptoms occurred at the age of 46 years during a physical examination, showing a bilateral positive Babinski sign. The patient suffered from concentration and cognitive problems over a few years, but an initial brain MRI was described as normal. A new MRI at the age of 56 years was performed due to a mild tetraspasticity and revealed atrophy and T2 signal alterations in the medulla oblongata and in the periventricular occipital white matter, as well as a frontoparietal cyst on the left side.

The patient exhibited depressive symptoms, reported dizziness, experienced recurrent syncopes, and a gait disorder (last GMFCS II). The patient experienced subjective respiratory distress when falling asleep and urge incontinence was reported. Upon physical examination, a slight gait ataxia was seen; these symptoms pushed the patient to quit their job.

The patient reported that her mother had suffered from dementia and a gait disorder at the age of 60 years. The patient’s own children were unaffected at that time. Patient 7 died of a stroke at the age of 63 years, presenting with hemiparesis and dysarthria, having the risk factors of a 30 pack-year smoking history, hypercholesterinemia, and arterial hypertension.

Patient 8 also showed a late onset of AxD. Dragging of the right leg was described as the first symptom, occurring at the age of 12 years. The patient developed orthostatic dysregulation and a general feeling of weakness. Dysphagia occurred, which was first considered as a psychosomatic symptom. The disease was discontinuously progressive, and the gait ataxia in particular worsened. This led to the necessity of a wheelchair and, after further progression, to dropping out of school at the age of 14 years. A reintegration was planned after the symptoms improved and the patient was able to walk again. Upon physical examination, a positive Babinski sign on the right foot was observed. Respiratory problems (gasping and a sensation of laryngeal constriction) described by the patient could not be objectively verified, and were therefore categorized as a part of a depressive mood disorder. The suspicion of Leigh syndrome occurred because of classical radiological features. In the MRI, focal demyelination in the medulla oblongata and the upper cervical cord were described.

This patient died suddenly at the age of 15 years; a resuscitation was not successful. The diagnosis of AxD was made post-mortem via brain autopsy, showing massive Rosenthal fibres.

**Figure 1:**
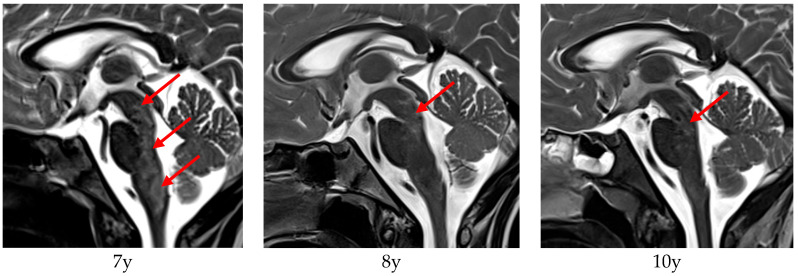
cMRI (T2) of patient 9 at the age of 7 years (1 month after first immunoglobulin and steroid pulse therapy), 8 years, and 10 years. The edematous T2 hyperintense brainstem lesions (red arrows) at the age of 7 years improved over time, correlating to clinical condition of the patient, who started with a massive loss of weight and dysarthria at onset of disease, evolving to a progressively stable condition.

Patient 9 showed fine motor skills dysfunction and balance issues beginning at the age of two years. Vomiting was observed at the age of six years and suspended after a short time, but dysphagia and weight loss occurred. Speech became dysarthric and nasal. Brain MRI showed periventricular cystic lesions and T2 signal alterations along the posterior brainstem, considered as a post-infectious rhombencephalitis. Pulse therapy with immunoglobulins and steroid lead to an improvement of the general condition and of eating and speaking. The diagnosis of AxD was confirmed at the age of seven years. At the age of almost eight years, the patient experienced deterioration in her general condition (dysphagia, dysarthria, sleep apnoea) and was hospitalized for another therapy trial with immunoglobulins and steroids, again with subsequent clinical improvements afterwards. Brain MRI revealed typical signs of AxD. Corresponding MRI images are shown in Figure 1, revealing a decrease in the brainstem T2 hyperintensities over time, correlating with the improved clinical condition between the onset of disease and last examination. In the most recent examination, the patient was in a stable clinical state with only minor symptoms.

In summary, all patients had at least temporarily motor function abnormalities, seven of them showing spasticity, and six of these presented with muscle hypotonia simultaneously. Seizures occurred in six patients, with an onset at different ages, the earliest being at the age of 11 months (patient 3) and the latest presenting with epileptic activity in EEG at the age of 21 years (patient 6).

Four (and one temporarily) out of nine patients suffered from macrocephaly; they were all classified as having an infantile form. One infantile patient was normocephalic.

Apnoea was a common symptom in our cohort, occurring in six patients, and two additional patients reported a subjective feeling of breathlessness (patient 8) or “breathing impairment” (patient 6), with the earliest onset of apnoea being at the age of one year.

Only two of our patients (1 and 6) shared the same pathogenic *GFAP* variant but showed rather different disease courses. Whereas both had an infantile disease onset, patient 1 was never able to walk alone and deteriorated early, with seizures and failure to thrive at the age of one year and bulbar symptoms before the fifth birthday. However, patient 6 had a relatively slow disease progression, with the temporary ability to walk in the first years of life and a gradual decline of motor capabilities over years. Seizures occurred in young adulthood. The last follow up was at the age of 33 years.

### 3.2. Steroids

Two patients received long-term or pulse corticosteroid treatment, which resulted in at least temporary improvements in their symptoms.

In patient 5, various corticosteroid regimens were used during episodes of acute exacerbation, such as methylprednisolone pulse therapy (20 mg/kg iv for 3–5 days) and different prednisolone protocols: (1) 0.4–2 mg/kg body weight for 1–2 days; (2) 40 mg (≈1.4 mg/kg body weight) daily for 7 days with a 5-week taper, followed by low-dose maintenance; and (3) repetitive 1 mg/kg body weight for 7 days every 21 days. At the age of 4 years and 2 months, a significant reduction in hemiparesis was observed within days after pulse therapy. At the age of 7 years and 6 months, spasticity and language deficits improved within days during treatment with prednisolone. At the age of 8 years, seizures decreased for 3 days after pulse therapy (regimen 1) and resolved two days after high-dose prednisolone treatment followed by a tapering (regimen 2). Later, the regime of 1 mg/kg for 7 days every 21 days (regimen 3) was preferred and seemed to combine clinical benefit with minimal side effects.

Patient 9 received IVIG for 5 days, followed by a 5-day course of intravenous corticosteroid pulse therapy (dose unspecified) at the age of 7 years and 6 months, under a presumptive diagnosis of autoimmune rhombencephalitis. Within days after treatment, symptoms like dysarthria, dysphonia, and dysphagia improved, and the patient resumed weight gain. At the age of 7 years and 11 months, due to worsening dysphagia, nocturnal dyspnea, and weakness, the regimen was repeated (IVIG for 2 days and corticosteroids for 5 days), again leading to further symptom improvement within days. As of the last follow-up, no further exacerbations have occurred and no additional treatments were needed.

### 3.3. Healthcare Situation

The reported healthcare situation of seven patients is summarized in Table 2; patients 7 and 8 were excluded due to the lack of data.

It is noteworthy that all of the seven patients, with an infantile or juvenile onset of disease, sooner or later reached a state of disability with a degree of disability between 50 and 100% and a level of care between 2 and 5. Most caregivers (6/7) received a payment from nursing insurance. All patients were categorized as completely helpless, were permitted to take a person with them on public transport, and had mobility (except from patient 9), or even severe mobility, problems (5/7). These categorizations are consistent with the high GMFCS scores of the patients listed in Table 1, except for patient 5, for whom the disability grade, the mark “aG”, and the level of care might have not been adjusted after the patient was able to walk again without his wheelchair. Despite the presence of motor deficits, none of the patients were deaf or blind. Six of these seven patients received preventive care and five had specialized outpatient pediatric palliative care.

The requirement of assistive devices was high but heterogenous. Only patient 9 did not need any assistive devices. Some patients required assistive devices only temporarily or during the course of the disease (patient 6), whereas others required multiple devices since childhood (as patient 3).

Consistent with their mobility issues, five (and one temporarily) of these seven patients used a wheelchair or rehabilitation cart. A G-tube was placed in five patients, one of whom required it only temporarily, reflecting the commonly observed dysphagia. Patient 9 did not need a G-tube despite the dysphagia. The need for orthoses was also common in our cohort (4/7); communication aids were used for only one patient, even though dysarthria was seen in almost all seven patients (except for patient 3, who never acquired language).

Almost all (6/7) patients needed some kind of integration assistance, early support, or an individual assistant for the improvement of social participation. While four patients were able to go to a regular school, two of them had to change school system after a decline in capabilities (patients 4 and 6); others were affected earlier and attended an integrative school (patient 2) or daycare from an early age (patients 1 and 3).

All patients received additional therapies, with physiotherapy being the most common (6/7) followed by logopaedia (4/7), reflecting the frequent occurrence of bulbar symptoms in our patients.

### 3.4. Summary of Patient Classification

The nine patients reported in this study were classified according to four different published classification systems (see Supplementary Tables S1–S4).

Among them, six patients presented with an infantile onset, one with a juvenile onset, and one with an adult onset of disease. The age of onset for patient 9 remains uncertain and could be either infantile or juvenile.

When classifying our patients according to the system proposed by Yoshida et al. (see Supplementary Table S1), five of the nine patients were categorized as intermediate type, as they met the core criteria for both cerebral and bulbospinal types. Two patients were classified as bulbospinal type. From the remaining two patients, one (patient 5) had clinical core features of both types but only MRI core features of the bulbospinal type. The other patient (patient 9) had MRI criteria of both types, but only clinical core features of the bulbospinal type. Thus, these patients cannot be strictly classified but may be considered the intermediate type [20].

Prust et al.’s classification (see Supplementary Table S2) divides patients into types 1 and 2. By application of this classification on our cohort, five patients were categorized as type 1, while two were classified as type 2. The remaining two patients showed significant overlap in clinical symptoms or MRI features, as detailed in Supplementary Table S2. Notably, (pseudo)bulbar symptoms, which are typically associated with type 2 in the Prust et al. system or with late-onset patients (Srivastava et al.), were observed in seven of the nine patients in our study, including all those with an early onset of disease (see Supplementary Tables S2 and S3) [6,21].

The classification by Mura et al. is based on the Prust et al. classification, but emphasizes disease progression, categorizing patients based on whether they experience a decline in capabilities (types 1a–c) or stable disease (types 1d and 2) [22,23]. Two of our patients were classified as type 1b, two as type 1c, and two as type 2. As reported, two patients showed lasting clinical improvement, which complicates their classification within this system. One other patient (2) demonstrated an atypical course of disease, with delayed autonomous ambulation but early deterioration.

Additionally, it is noteworthy that all of our patients, including those with late-onset disease, fulfilled several MRI criteria considered typical for AxD, as defined by van der Knaap et al. [9]. These findings are generally considered to be more characteristic for early-onset patients, as described in the traditional classification [6], or for type 1 patients, as described in the Prust et al. classification [21].

### 3.5. Alexander Disease Severity Score (ADSS)

Existing classification systems are complex and do not allow quantification of disease severity at different time points. For future studies, it may be beneficial to implement a severity score to evaluate the impact of new therapeutic approaches or experimental treatments. In this study, we propose a severity score based on six key symptoms—feeding problems, epilepsy, motor dysfunction, failure to thrive, language impairments, and apnoea—all of which are potentially life threatening, or have a significant effect on quality of life, and may occur at any age. This severity score reflects the clinical condition of patients at a specific time point. We categorized our patients by this score based on the reported clinical findings (see Table 3 and Table 4).

We then classified our patients accordingly.

Before clinical implementation of the ADSS, validation in a larger cohort of AxD patients will be necessary. Given the rarity of AxD, we performed a brief power calculation to determine the number of participants required for future validation studies. We expect the ADSS to vary with a standard deviation of sd = 3.67. With an alpha error of 5% and a power of 80%, a clinically relevant difference of 2 (3, 4) would require *n* = 54 (25, 15) subjects per group in a two-group comparison.

### 3.6. Genetic Findings

Four out of eight patients with known genetic variants harbour pathogenic variants in the hotspot codon Arg79 of the *GFAP* gene [21,34].

We identified three *GFAP* gene variants, which had not been published to date: c.1118A>G (p.Glu373Gly) and c.1013T>C (p.Leu338Pro), both in exon 6, (the latter is described by A. Messing as an unpublished variant, without neuropathological confirmation [27]), and c.209G>C (p.Arg70Pro) in exon 1. These three variants were all present in a heterozygous state. They were predicted to be non-truncating on the protein level but leading to non-synonymous amino acids alteration located in mutational hotspot regions [30]. Two of these variants arose de novo, while for the third (c.209G>C, patient 7), no segregation data was available. However, the patient’s mother developed dementia and gait disorders at the age of 60 years. According to ACMG guidelines, the variants c.1118A>G (PM1, PP2, PM2, PM5, PP3, PS2) and c.1013T>C (PM1, PP2, PM2, PP3, PS2) were classified as pathogenic variants, while the variant c.209G>C was classified as likely pathogenic (PM1, PP2, PM2, PM5, PP3) [30]. Notably, Arg70 is recognized as a hotspot codon for adult-onset AxD and an alteration affecting this codon was observed in patient 7.

In addition to the pathogenic variant c.1118A>G, patient 4 also carried another rare variant (c.170C>T, p.Ala57Val). As this variant was also present in the patient’s healthy father, we classified this variant as likely non-pathogenic.

The genetic testing of patient 3 also revealed the microdeletion 15p13.2q13.3. This genetic alteration can also be responsible for neurodevelopmental and neuropsychiatric conditions, as well as speech problems and epilepsy, but also shows a variable clinical course and incomplete penetrance [35]. The *GFAP* variant of the patient is described before and would be sufficient to explain all the symptoms [26].

## 4. Discussion

We described the clinical course of nine patients with AxD, including diagnostic and treatment approaches, as well as radiologic features. The application of existing classification systems posed to be challenging for atypical cases with signs of clinical improvements. To better assess intra- and inter-individual disease severity, the Alexander Disease Severity Score (ADSS) could be a useful tool.

The nine cases reported here highlight the broad clinical spectrum of AxD. The age of onset ranged from four months to 46 years. Notably, at least intermittent motor function abnormalities were reported in all patients (Table 1). Pseudobulbar signs, spasticity, and muscular hypotonia were common, each observed in seven of these patients.

Macrocephaly, considered a hallmark of early-onset AxD patients, was only observed in four (and one temporary) of nine patients. All patients with macrocephaly had a disease onset at or before 12 months of age. Interestingly, one patient (1) with an onset at four months of age, and patient 9 with the unclear onset at maybe 2 or 6 years of age, did not present with macrocephaly over time, suggesting that there may not be a robust correlation between early onset and macrocephaly in our cohort.

Additionally, apnoea occurred in our patients at young ages, which underscores the importance of polysomnography for early detection, despite its typical description as a symptom primarily seen in adult patients [6].

Corticosteroids are known to have both anti-inflammatory and pro-inflammatory effects. They reduce brain edema by decreasing blood–brain barrier permeability and inhibiting active sodium transport in brain capillaries [36,37]. Kora et al. observed a temporary reduction in inflammatory cytokines following steroid treatment [16]. Brain swelling is commonly observed in MRI examinations of patients with AxD [9]. Microglia and astrocytes play significant roles in the pathogenesis of AxD [38,39].

While corticosteroids are effective in reducing inflammation and managing edema, their mechanisms in modulating protein aggregates such as Rosenthal fibres, as well as their influence on microglia and astrocytes, remain unclear. Further research is necessary to elucidate the impact of corticosteroids on these specific pathophysiological features.

In our cohort, two AxD patients were treated with corticosteroids for varying dosages and durations; both showed at least temporary clinical improvement following steroid therapy. In patient 5, different corticosteroid administration regimens were used. Precise dosage information and exact timelines of treatment responses were not available for all cases. Because of the retrospective design, the absence of standardized outcome measures, and the very small sample size, we report only observational findings without intending to provide definitive evidence of causality. It remains uncertain whether spontaneous symptom fluctuations associated with the natural course of the disease contributed to the observed improvement. Temporary symptom improvement with corticosteroid treatment has also been reported in two cases in the literature [16,17]. However, to date, no studies have systematically analyzed the use of corticosteroids in AxD. Therefore, corticosteroids should only be considered as an experimental treatment option, particularly given the lack of available causative therapies for AxD. Future research with standardized protocols and outcome measures in a multicentric study will be required to assess the effectiveness of steroids in AxD patients.

Patient 9 demonstrated not only clinical improvement but also radiological signs of decreased signal abnormalities of the medulla oblongata on MRI. To our knowledge, this has not been previously reported in any other case of AxD. However, as MRI scans were not performed immediately before and after steroid and immunoglobulin pulse therapy, it remains unclear whether the observed improvements were solely due to the immunomodulatory treatment or if they might reflect spontaneous fluctuations.

It remains elusive whether the treatment with dimethyl fumarate in patient 6 had stabilizing effects and whether the reported side effects were related to the trial.

It is worth noting that other authors have reported successful experimental symptom management with alternative medications. For instance, Sechi et al. described the halting and even reversal of symptoms in an adult-onset AxD patient following ceftriaxone treatment, with a four-year follow-up [40,41]. Similarly, a case report from 2006 highlighted the positive effects of Thyrotropin-Releasing Hormone (TRH) treatment [42]. In a nationwide survey by Yoshida et al., TRH was tested in three patients, with one patient showing improvement in ataxia and certain brainstem abnormalities [20]. However, none of our patients received these treatments.

Recently, antisense oligonucleotides (ASO) have been proposed as a potential treatment option for AxD [15]. A clinical study led by the pharmaceutical company IONIS is currently in progress, investigating the use of antisense oligonucleotide targeting GFAP mRNA, referred to as ION373. This treatment has been designated as an orphan drug by the European Medicines Agency (EMA) since 2020. The goal is to halt disease progression by reducing GFAP mRNA levels, thereby decreasing GFAP protein production and mitigating its toxic accumulation [43,44].

Over the past decades, several well-established and valuable classification systems for patients with AxD have been published. Based on age at onset, specific symptoms, and clinical as well as diagnostic findings, patients are attributed to different forms of the disease: cerebral, bulbospinal, or intermittent type according to Yoshida et al. [20]; type 1 or 2 as classified by Prust et al. [21]; and infantile, juvenile, or adult type as described by Srivastava et al. [6]. In addition, Mura et al. [22] and Vaia et al. [23] categorized AxD patients into subgroups 1a, 1b, 1c, and 1d based on the evolution over time focused on neuromotor development and neurological deterioration. These classifications allow, to some extent, a preview of the expected course. However, the highly variable course of disease, especially in the infantile form of AxD, can be difficult to capture within the existing classification systems.

Similar variability and difficulty occur in our cohort, which includes patients with atypical slow progression (e.g., patient 6) and even clinical improvement (e.g., patients 5 and 9) following steroid therapy. Notably, patient 5 exhibited an atypical course, initially presenting with severe symptoms, including respiratory insufficiency requiring resuscitation, but had subsequently regained the ability to walk independently. These patients have now reached ages between 9 and 33 years after an early onset of the disease. Another patient with an infantile onset of AxD and a life expectancy of 39 years has been described in the literature [45].

It is also noteworthy that in our cohort, bulbar symptoms, typically associated with type 2 or late-onset disease, were present in seven of nine patients; however, six of these seven patients (or seven if patient 9 is classified as early-onset) had an early-onset disease and would instead be classified as type 1 [6,21] (see Supplementary Tables S2–S4). These examples reveal a limitation of current systems in assigning some patients to specific AxD subtypes. Furthermore, existing classification systems do not capture disease severity at a specific time point—an important gap, as symptoms may improve with current or future therapeutic interventions.

To address this, we propose the Alexander Disease Severity Score (ADSS), a scoring system based on the severity of six clinical key features consistently observed across age groups and subtypes: feeding problems, epilepsy, motor function, failure to thrive, language impairment, and apnoea (Table 3). The total score may serve as a time point-specific surrogate measure of disease burden, describing the severity of impairment (mildly, moderately, or severely affected). ADSS could be applied universally to all AxD patients, regardless of age or disease subtype.

Importantly, several of these features improved in some of our patients following steroid therapy. Experimental treatments reported in the literature have also demonstrated benefits for these features. One patient treated with ceftriaxone showed improvements in motor and language function [40,41], while prednisolone therapy positively affected vomiting and weight loss in a late-onset case [17], as well as a decrease in seizure frequency in an infantile case [16]. Therefore, the proposed score may be able to reflect changes in disease severity after therapeutic interventions.

If validated and incorporated into clinical practice and research, the ADSS could enhance intra- and interindividual comparisons of disease severity, thereby improving the evaluation of treatment study efforts. Similar scoring systems have been developed for other childhood neurodegenerative diseases, such as GM2 gangliosidosis [46] and Canavan leukodystrophy [47]. In the case of Canavan disease, these scoring systems are utilized in treatment trials [48,49].

Before clinical implementation of the ADSS, validation in a larger cohort of AxD patients will be necessary.

To validate the ADSS, independent assessments of the same patients by multiple clinicians are necessary to establish inter-rater reliability. We anticipate a high level of reliability, as the individual items (Table 3) allow minimal opportunity for subjective interpretation. To assess the convergent validity, the ADSS could be correlated with established functional and quality of life scales. Strong correlations would support its validity in reflecting disease severity. To evaluate the responsiveness of the ADSS to change and its sensitivity to clinical improvements or deterioration post-treatment, longitudinal studies will be required. Validation of the ADSS should preferably be conducted through multicenter studies.

The wide spectrum of symptoms observed in our cohort is also evident in the genotype–phenotype correlation. Despite four of the eight patients in our cohort carrying variants in the hotspot codon Arg79 [21,34], we observed considerable phenotypic differences, consistent with descriptions in the literature. For some variants, a correlation has been suggested, with variants affecting Arg239, for instance, being associated with a more severe phenotype, typically presenting in infancy, though not universally [6,50]. A variant affecting Arg79 did not result in identical disease expressions in our cohort (e.g., patient 1, with early onset and severe progression, compared to patient 6, with a very prolonged and mild course). This variability was evident across all age-of-onset groups [6]. It remains unclear which epigenetic or modifying factors contribute to this considerable variability in disease course.

In conclusion, AxD exhibits significant clinical variability, as reflected by the small cohort of patients reported here. Biomarkers like GFAP, as shown in mice and humans [51,52], may contribute to more accurate predictions of disease course in the future.

We also evaluated the care needs of our AxD patients, as reflected by the use of aids and support services within the German social care system. Our findings demonstrate that the level of required support varies significantly, although most patients need considerable assistance. In milder disease forms, patients require fewer devices and therapies; however, care needs may increase over time. It is important to note that this analysis provides a cross-sectional perspective, reflecting each patient’s needs at a single time point, with patients being at different stages of disease progression. All patients, except those with late-onset disease, were issued disability cards with the designation “helplessness”. This underscores the importance of early consultation with caregivers regarding available support options, as well as the need for regular reassessment to adapt therapies and devices as disease progression occurs. The considerable variability in healthcare needs emphasizes the clinical heterogeneity of AxD. While our findings are based on the German healthcare system, the observed variability in support requirements likely reflects broader, internationally relevant principles. The extent of assistance, use of assistive devices, and social participation needs may vary depending on the healthcare infrastructure, funding mechanisms, and policies of each country. Although Germany provides comprehensive insurance-based support, resource limitations in other countries may impact access to therapies and devices. This is exemplified in Germany by the coverage of assistive devices such as wheelchairs, as well as interventions such as physiotherapy and integration aids, to mitigate disadvantages—measures that are not universally accessible to all affected individuals across different countries worldwide. Nonetheless, the importance of early diagnosis, individualized multidisciplinary care, and periodic re-evaluation of patient needs is consistent across healthcare systems. Promoting awareness, developing adaptable care protocols, and supporting inclusive educational policies are crucial undertakings worldwide. These principles highlight the need for international collaboration to improve quality of life and social inclusion for individuals with AxD, regardless of geographic location.

### Limitations

The study’s primary limitation is the small sample size of nine patients, which restricts the generalizability of findings. Before clinical implementation of the proposed Alexander Disease Severity Score (ADSS), validation of this score in a larger cohort is required. Limitations also apply to the interpretation of steroid therapy effects, which we report in only two patients after extraction and analysis of retrospective data from medical records. Assessment at a single time point only captures a fraction of the overall disease course and the patient’s condition. We also cannot distinguish with certainty if some of the symptoms of patient 3 are aggravated by the microdeletion. From case 5, we only have the genetic finding described in physician’s letters, not the original result of the laboratory. We only had access to the descriptions and diagnostic reports of MRI pictures of patients 6 and 8 and used them for classification

## 5. Conclusions

Alexander disease is a severe and progressively disabling leukodystrophy. In this study, we contribute additional phenotypic data to the existing literature, propose a novel severity score based on clinical key parameters, report three previously unpublished pathogenic gene variants, and summarize the heterogeneity of healthcare support required by affected individuals.

Once AxD is diagnosed using an MRI scan or genetic testing, it is important to inform caregivers about the broad clinical spectrum of AxD, including potentially life-threatening manifestations such as apnoea. Polysomnography should be considered to monitor this symptom. Caregivers should be educated regarding the substantial, though heterogeneous, need for care and support. Currently, therapeutic approaches focus on optimized supportive care, which varies depending on disease severity. In the event of clinical deterioration, individualized experimental treatments such as steroids may be considered, pending the approval of more definitive therapies. While classification into existing systems may be challenging, the proposed severity score can be easily assessed at each visit, offering a more practical and reproducible tool for monitoring disease progression.

## Figures and Tables

**Table 1:** Summary of genetic variants, MRI findings and most frequent symptoms according to the time of first appearance or last examination; given ages are the onset time points, rounded to full years (except ages < 1 y). Legend: Rf = Rosenthal fibres; x = feature is present; - = feature is absent; m = months; y = years; † = deceased; temp. = temporary; a.s. = after stroke; d. = dementia; NA = not applicable; * = variant is described in the table by A. Messing as an unpublished variant without neuropathological confirmation [27]; ** = regressive; grey diagonally dashed = pictures not reviewed.

		Patient	1	2	3	4	5	6	7	8	9
general information		age at first symptoms	4m	6m	4m	10m	7m	1y	46y	12y	2y/6y
		age at last information/death	7y	9y	3y	13y	9y	33y	63y †	15y †	10y
		confirmation of diagnosis/*GFAP* gene variant	c.235C>T p.Arg79Cys	c.1126C>T p.Arg376Trp	c.235C>G p.Arg79Gly	c.1118A>G p.Glu373Gly	c.1013T>C p.Leu338Pro	c.235C>T p.Arg79Cys	c.209G>C p.Arg70Pro	autopsy: RF	c.236G>A p.Arg79His
		unpublished	-	-	-	x	X *	-	x	-	-
		steroids used and positive effect reported				-	x	-			x
symptoms	motor symptoms	motor function abnormality	4m	1y	x	9y	x	1y	47y	12y	temp.
		worst GMFCS (age)	V (6y)	IV–V (9y)	V (2y)	V (13y)	III	V (33y)	II (57y)	III (14y)	I (10y)
		spasticity	1y	x	x	10y	x	x	56y		-
		muscle hypotonia	3y	5y	1y	10y	7m	2y	-		temp. (8y)
		scoliosis	3y	-		x	-	13y	x		-
	brain stem symptoms	(pseudo)bulbar signs	4y	x	2y	10y	1y	x	a.s.	unclear	x
		vomiting	1m	9y	x	x	2y		-	-	x
		apnoea	5y	-	1y	13y	3y		53y		8y
		spastic paraparesis		-			temp. (7y)	x	x		-
		pyramidal signs	3y	9y	3y	10y	7y	11y	47y	14y	-
		ataxia	-	1y	-	10y	8y		57y	x	10y
		ocular movement abnormalities	1y	-	-	-	temp.	x	-	x	-
	other symptoms	underweight (<3 P.)	1y	1y	-	x	temp (1y)	-	-	-	temp. (6y)
		short stature (<3 P.)	3y	1y	temp. (2y)	-	-	-	-	-	temp. (8y)
		autonomic dysfunction	x	9y	x	x	-	14y	50y	12y	x
		psychomotor developmental delay/mental retardation	x	x	x	x	x	x	d.		-
		cognitive abnormalities	1y	-	x	x	-	7y	47y	-	-
		sleep disturbance	temp.	x	3y	-	-	x	x		temp. (9y)
		seizures	1y	2y	11m	3y	6y	x	-	-	-
		macrocephaly	-	3y	1y	x	temp.	9y	-	-	-
MRI	typical findings (van der Knaap et al., 2001) [9]	extensive cerebral white matter abnormalities with a frontal preponderance, either in the extent of the white matter abnormalities, the degree of swelling, the degree of signal change, or the degree of tissue loss (white matter atrophy or cystic degeneration)	1y, 5y	2y, 4y	x	10y		x			7y, 8y
		presence of a periventricular rim of decreased signal intensity on T2-weighted images and elevated signal intensity on T1-weighted images	5y	2y, 4y	x	10y		x			7y, 8y
		abnormalities of the basal ganglia and thalami, either in the form of elevated signal intensity and some swelling or of atrophy and elevated or decreased signal intensity on T2-weighted images	1y, 5y	2y, 4y	x	10a	2y	x			7y, 8y
		brain stem abnormalities, in particular involving the midbrain and medulla	5y	2y, 4y	x		2y	x	56y	13y, 15y	7y, 8y
		contrast enhancement involving one or more of the following structures: ventricular lining, periventricular rim of tissue, white matter of the frontal lobes, optic chiasm, fornix, basal ganglia, thalamus, dentate nucleus, and brain stem structures	5y	NA	NA		2y	x	NA		7y, 8y
	atypical findings	(Prust et al., 2011) [21] spinal cord atrophy (incomplete data)						x	x		
		cerebellar atrophy		x							
		brainstem atrophy						x	56y		
		predominance of posterior fossa white matter abnormalities							56y		
		contrast enhancement in posterior fossa structures		NA	NA				NA		x
		mass-like brainstem lesions	5y								7y, 8y **

**Table 2:** Disability degree and healthcare situation. Information based on a questionnaire. There is a lack of data for patient 7 and 8. Legend: - = no; x = yes; bw. = borrowed; temp. = temporary; pl. = planned; * = because of deterioration a change in school was necessary. Level of care (1 to 5): low impairment of independence (level 1) to severe impairment with special needs of care (level 5), associated with different levels of financial support.

Patient	1	2	3	4	5	6	9
disability card	x	x	x	x	x	x	x
degree of disability %	100	100	100	100	80	100	50
Bl—blind	-	-	-	-	-	-	-
B—accompanying person	x	x	x	-	-	x	-
G—mobility problems	x	x	x	x	x	x	-
aG—severe mobility problems	x	-	x	x	x	x	-
Gl—deaf	-	-	-	-	-	-	-
H—helpless	x	x	x	x	x	x	x
RF—leave of broadcasting fee	-	-	x	-	-	-	-
level of care	4	2	5	5	4	5	3
nursing insurance—payment	x	x	x	x	x		x
additional supportive care by impairment of daily routines	-	x		x	-		-
preventive care	x	x	x	x	x		x
**assistive devices**	x	x	x	x	x	x	-
wheelchair	-	-	-	-	temp.	x	-
wheelchair with adjusted seat	-	-	-	x	-		-
rehab cart	x	bw.	x	x	-		-
showerchair	-	-	x	x	-	x	-
toiletchair	-	-	-	x	-		-
lift	-	-	-	x	-	x	-
positioning aids	-	-	x	-	-		-
therapychair	x	-	x	-	-		-
bike with wheelchair—Rollfiets	-		-	-	-		-
ortheses	x	-	x	x	x		-
communcation aid	-	-	-	x	-		-
nursing bed	x	-	x	x	-	x	-
incontinence supply		-	x	x	-		-
g-tube	x	-	x	x	temp.	x	-
other devices	x	-	x	x	x		-
integration assistence	x	x	x	x	x		x
early support	x		x	-	-		-
individual helper	pl.	x	-	x	x		x
specialized outpatient paediatric palliative care	x	-	x	x	-	x	x
hospice visits	x	-		x	-		-
charity support		x	x	x	-		-
**therapies**	x	x	x	x	x	x	x
physiotherapy	x	x	x	x	x	x	-
occupational therapy	-	x	-	-	x		-
logopeadia	x	-	x	x	-		x
hippotherapy	-	x	-	x	-		-
other	x	-	-	-	x		x
daycare/school	x	x	x	x	x	x	x
regular daycare	-	-	-	-	x		-
integrative daycare	x	-	x	-	-		-
regular school	-	-	-	x *	x	x *	x
integrative school	-	x	-	-	-		-
school for children with special needs	-	-	-	x *	-	x *	-
driving service	-	x	x	x	x		-

**Table 3:** Alexander Disease Severity Score (ADSS) definition of items: 0–12 points possible, mild disease; 0–4 points, moderate disease; 5–8 points, severe disease; 9–12 points. * Described in physicians letter or in polysomnography. ** Steroids given as ICISS scheme or in higher doses (pulse therapy) are rated as antiepileptic treatment; long-term low-dose therapy is not rated as AED (antiepileptic drug). Percentiles determined and defined according to KiGGS [24].

Category	Deficits Absent/ Within Normal Limits (0P)	Deficits Present Intermittently or Mild (1P)	Deficits Present Constantly or Pronounced (2P)
feeding problems	no	yes	tube feeding necessary
epilepsy during the last year	no epileptic seizures, not taking antiepileptic drug/s (AED) **	1–2 unprovoked seizures/year, not taking AED/s OR no seizures but on 1 AED **	≥3 unprovoked seizures /year OR no seizures but requires more than 1 AED ** OR taking one sufficient AED + having seizures
motor function appropriate to age	yes (i.e., GMFCS 1)	no, mild deficits (i.e., GMFCS 2-3)	no, pronounced deficits (i.e., GMFCS 4–5)
failure to thrive (poor weight and height gain)	no	no dystrophy (>3. percentile) but crossing of two main percentiles (5, 10, 25, 50, 75, 90, 95)	<3. percentile
language appropriate to age	yes	mildy affected (i.e., verbal skills sufficient for communication with strangers)	severely affected (only family can understand)
apnoea *	no	history of apnoea, not in the last 3 years OR only clinical suspicion because of patients/parents reports	yes

**Table 4:** Classification of our patients according to the Alexander Disease Severity Score (ADSS) at most recent presentation/ examination (patient 7 at last examination before stroke). Patients 5, 7, 8, and 9 were mildly affected, patients 2 and 6 were moderately affected, and patients 1, 3, and 4 had a severe form of disease at the time of examination.

Patient	1	2	3	4	5	6	7	8	9
feeding problems	2	1	2	2	0	2	0	1	0
epilepsy	2	1	2	2	2	2	0	0	0
motor function appropriate to age	2	2	2	2	1	2	1	2	0
failure to thrive	2	2	0	1	0	0	0	0	0
language appropriate to age	2	1	2	2	0	2	0	0	1
apnoea	2	0	1	2	1	1	1	1	2
severity score	12	7	9	11	4	9	2	4	3

## Data Availability

The data presented in this study are not available in a more detailed form due to data protection reasons.

## Supplementary Materials

The following supporting information can be downloaded at https://www.mdpi.com/article/10.3390/jcm14196918/s1: Table S1: Classification of the cases according to Yoshida et al. [20] focused on core features; Table S2: Summary of symptoms and MRI findings, focusing on the Prust et al. classification [21]; Table S3: Summary of symptoms and MRI findings, focusing on traditional classification [9]; Table S4: Classification of the cases according to Mura et al. and Vaia et al. [22,23].

## Abbreviations

The following abbreviations are used in this manuscript:

AxD	Alexander disease
CNS	Central nervous system
CT	Computed tomography
ECG	Electrocardiogram
EEG	Electroencephalography
EMA	European medicines agency
EMG	Electromyography
ENG	Electroneurography
GFAP	Glial fibrillary acidic protein
GMFCS	Gross motor function classification system
G-Tube	Gastrostomy tube
HGMD	Human gene mutation database
HGVS	Human genome variation society
HSP27	Heat shock protein 27
IVIG	Intravenous immunoglobulins
MRI	Magnetic resonance imaging
Nrf2	Nuclear factor erythroid 2-related factor 2
RF	Rosenthal fibres
