# Supplementary material for: Description of the Hamburg Alexander Leukodystrophy Cohort—Insights into Practical Classification and the Care Situation
Source: J Clin Med. 2025 Sep 29;14(19):6918. doi: 10.3390/jcm14196918 (PMC12524457; doi:10.3390/jcm14196918)
Supplement: Supplementary file 1 [file jcm-14-06918-s001.zip › jcm-3794250-supplementary.pdf]

Supplements:

Supplement Table S1: Classification of the cases according to Yoshida et al. focused on core features

		patient	1	2	3	4	5	6	7	8	9
core features	cerebral (type 1)	psychomotor developmental delay/ mental retardation	x	x	x	x	x	x	d.		-
		convulsions	1y	2y	11m	3y	6y	x	-	-	-
		macrocephaly	-	3y	1y	x	temp.	9y	-	-	-
		MRI: cerebral white matter abnormalities especially frontal	x	x	x	x		x			x
	bulbospinal (type 2)	muscle weakness		7y		10y	temp. (4y)	10y	-	-	-
		hyperreflexia (sometimes a-/hyporeflexia)		x	2y (hypo.)	x	x	x	x	x	-
		pos. Babinski sign	3y	9y	3y	10y	7y	x	47y	14y	-
		dysarthria, dysphonia, dysphagia	4y	x	2y	10y	1y	x	a.s.	unclear	x
		MRI: medulla/ cervical atrophy or signal change	x	x	x	x	x	x	x		x
	intermediate (type 3)	clinical core features of both	x	x	x	x	x	x	-	-	-
		MRI core features of both	x	x	x	x	-	x	-	-	x
	type		3	3	3	3	2/3	3	2	2	2/3

Supplement Table S1: Classification according to Yoshida et al. [1] only core features given; given ages are the onset time points, rounded to full years (except ages <1y)

Legend: x = feature is present; - = feature is absent; m = months; y = years; temp. = temporary; a.s.= after stroke; d. = dementia; grey diagonally dashed = pictures not reviewed; grey background = typical for bulbospinal type

Supplement Table S2: Summary of symptoms and MRI findings focusing on the Prust et al. classification

		patient	1	2	3	4	5	6	7	8	9
type 1 Prust et al 2001	symptoms (Prust et al. 2011)	early age of onset (<4y)	x	x	x	x	x	x	-	-	- / x
		seizures	1y	2y	11m	3y	6y	x	-	-	-
		macrocephaly	-	3y	1y	x	temp.	9y	-	-	-
		failure to thrive (lack of normal weight and height gains)	3y	1y	-	-	-	-	-	-	temp. (8y)
		encephalopathy	-	2y	x	x	x	8y	x	x	-
		motor development delay	x	1y	x	x	-	1y	-	-	-
		developmental delay	x	x	x	x	x	x	-		-
		paroxysmal deterioration	3y		x	x	7y			x	
	MRI typical findings (van der Knaap et al. 2001)	extensive cerebral white matter abnormalities with a frontal preponderance, either in the extent of the white matter abnormalities, the degree of swelling, the degree of signal change, or the degree of tissue loss (white matter atrophy or cystic degeneration)	x	x	x	x		x			x
		presence of a periventricular rim of decreased signal intensity on T2-weighted images and elevated signal intensity on T1-weighted images	x	x	x	x		x			x
		abnormalities of the basal ganglia and thalami, either in the form of elevated signal intensity and some swelling or of atrophy and elevated or decreased signal intensity on T2-weighted images	x	x	x	x	x	x			x
		brain stem abnormalities, in particular involving the midbrain and medulla	x	x	x		x	x	x	x	x
		contrast enhancement involving one or more of the following structures: ventricular lining, periventricular rim of tissue, white matter of the frontal lobes, optic chiasm, fornix, basal ganglia, thalamus, dentate nucleus, and brain stem structures	x	NA	NA		x	x	NA		x
type 2 Prust et al 2011	symptoms (Prust et al. 2011)	bulbar symptoms	4y	x	2y	10y	1y	x	a.s.	unclear	x
		ocular movement abnormalities	1y	-	-	-	temp.	x	-	x	-
		autonomic dysfunction	x	9y	x	x	-	14y	50y	12y	x
		development of gait disturbance	NA	x	NA	x	4y	2y	x	x	-
		lack of cognitive dysfunction	-	x	-	-	x	-	-	x	x
		palatal myoclonus									
	MRI atypical findings (Prust et al. 2011)	spinal cord atrophy (incomplete data)						x	x		
		cerebellar atrophy		x							
		brainstem atrophy						x	x		
		predominance of posterior fossa white matter abnormalities							x		
type			1	1	1	1	1	1/2	2	2	1/2

Supplement Table S2: Classification according to Prust et al. [2]; given ages are the onset time points, rounded to full years (except ages <1y)

Legend: x = feature is present; - = feature is absent; m = months; y = years; temp. = temporary; a.s. = after stroke; NA = not applicable; grey diagonally dashed = pictures not reviewed; grey background = typical for type 1

Supplement Table S3: Summary of symptoms and MRI findings, focusing on traditional classification

		patient	1	2	3	4	5	6	7	8	9
typical for infantile form	clinical symptoms	infantile onset	4m	6m	4m	10m	7m	1y	-	-	2y/ 6y
		macrocephaly	-	3y	1y	x	temp.	9y	-	-	-
		seizures	1y	2y	11m	3y	6y	x	-	-	-
		developmental delay	x	x	x	x	x	x	-		-
		dysarthria	x	x	NA	x	1y	x	a.s.	-	x
		failure to thrive (poor weight gain)	1y	1y	-	x	temp (1y)	-	-	-	temp (6y)
	MRI	T2-hypointense periventricular rim	x	x	x	x		x			x
		symmetric signal abnormality of medulla	-	x	x	x	x		x		both sides
		basal ganglia or thalami involvement	x	x	x	x	x	x			x
		T2 hyperintensity, especially frontal white matter	x	x	x	x		x			x
typical for juvenile form	clinical symptoms	juvenile onset	-	-	-	-	-	-	-	12y	2y/6y
		scoliosis	3y	-		x	-	13y	x		-
		short stature	3y	9y	temp. (3y)	x	-	-	-	-	-
		vomiting	1m	9y	x	x	2y		-	-	x
		failure to thrive (poor weight gain)	1y	1y	-	x	1y	-	-	-	6y
		(pseudo)bulbar sign	4y	x	2y	10y	1y	x	a.s.	x	x
		autonomic dysfunction	x	9y	x	x	-	14y	50y	12y	x
	MRI	mass-like brainstem lesions	x		-						temp.
		contrast enhancement in posterior fossa structures									x
		T2 hyperintensity in cerebellar white matter or hilus of dentate nuclei	x	x		x			x		x
typical for adult form	clinical symptoms	adult onset	-	-	-	-	-	-	46y	-	-
		gait disturbance	NA	x	NA	x	x	x	x	x	-
		spastic paraparesis		-			temp. (7y)	x	x		-
		pyramidal signs	3y	9y	3y	10y	7y	x	47y	14y	-
		ataxia	-	1y	-	10y	8y		57y	x	10y
		sleep apnoea	5y	-	1y	13y	3y		53y		8y
		normocephaly	x	-	-	-	x	-	x	x	x
		palatal myoclonus									
		ocular movement abnormalities	1y	-	-	-	temp.	x	-	x	-
		autonomic dysfunction	x	9y	x	x	-	14y	50y	12y	x
		(pseudo)bulbar signs	4y	x	2y	10y	1y	x	a.s.	unclear	x
	MRI	medullary and cervical cord atrophy (incomplete data)						x	x		
		T2 hyperintensity in cerebellar white matter or hilus of dentate nuclei	x	x		x			x		x
type			inf.	inf.	inf.	inf.	inf.	inf.	adult	juv.	inf./juv.

Supplement Table S3: Classification according to the traditional classification focused on the updated version by Srivastava et al. [3]; given ages are the onset time points, rounded to full years (except ages <1y)

Legend: x = feature is present; - = feature is absent; m = months; y = years; temp. = temporary; a.s. = after stroke; inf. = infantile type; juv. = juvenile; grey diagonally dashed = pictures not reviewed; grey background = typical for infantile onset; vertical dashed = typical for juvenile onset; horizontal dashed = typical for adult onset

Supplement Table S4: Classification of the cases according to Mura et al. and Vaia et al.

	patient	1	2	3	4	5	6	7	8	9
neuromotor development	developmental delay	x	x	x	x	x	x	-		-
	no postural acquisition (type 1a)	-	-	-	-	-	-	-	-	-
	no autonomous ambulation (type 1b)	x	-	x	-	-	-	-	-	-
	delayed autonomous ambulation (type 1c/1d)	-	x	-	x	-	x	-	-	-
	autonomous ambulation from regular age (type 2)	-	-	-	-	x	-	x	x	x
neurologic deterioration	early fatal course (type 1a)	-	-	-	-	-	-	-	-	-
	deterioration <6y (type 1b)	x	x	x	-	*	-	-	-	-
	deterioration >6y (type 1c)	-	-	-	x	-	x	-	-	*
	stable beyond beginning of adolescence (type 1d)	-	-	-	-	*	-	x	x	*
	**type**	1b	1b/1c	1b	1c	1b/2	1c	2	2	1c/1d/2

Supplement Table S4: Classification according to Mura et al. and Vaia et al. [4, 5];

Legend: x = feature is present; - = feature is absent; * = unclear to classify because of deterioration but also clinical improvement over time

1. Yoshida T, Sasaki M, Yoshida M, Namekawa M, Okamoto Y, Tsujino S, et al. Nationwide survey of Alexander disease in Japan and proposed new guidelines for diagnosis. J Neurol. 2011;258(11):1998-2008.
2. Prust M, Wang J, Morizono H, Messing A, Brenner M, Gordon E, et al. GFAP mutations, age at onset, and clinical subtypes in Alexander disease. Neurology. 2011;77(13):1287-94.
3. Srivastava S, Waldman A, Naidu S. Alexander Disease. 2002 15.11.2002 [updated 12.11.2020] [cited 15.06.2025]. In: GeneReviews((R)) [Internet]. Seattle (WA): University of Washington, [cited 15.06.2025]. Available from: https://www.ncbi.nlm.nih.gov/pubmed/20301351.
4. Vaia Y, Mura E, Tonduti D. Type I Alexander disease: Update and validation of the clinical evolution-based classification. Mol Genet Metab. 2023;138(3):107540.
5. Mura E, Nicita F, Masnada S, Battini R, Ticci C, Montomoli M, et al. Alexander disease evolution over time: data from an Italian cohort of pediatric-onset patients. Mol Genet Metab. 2021;134(4):353-8.
